# High concentrations of middle ear antimicrobial peptides and proteins and proinflammatory cytokines are associated with detection of middle ear pathogens in children with recurrent acute otitis media

**DOI:** 10.1371/journal.pone.0227080

**Published:** 2019-12-26

**Authors:** Elke J. Seppanen, Ruth B. Thornton, Karli J. Corscadden, Caitlyn M. Granland, Julie Hibbert, Angela Fuery, Selma P. Wiertsema, Shyan Vijayasekaran, Harvey L. Coates, Peter Jacoby, Andrew Currie, Peter C. Richmond, Lea-Ann S. Kirkham

**Affiliations:** 1 Wesfarmers Centre of Vaccines and Infectious Diseases, Telethon Kids Institute, Perth, Western Australia, Australia; 2 School of Biomedical Sciences, Faculty Health and Medical Science, University of Western Australia, Perth, Western Australia, Australia; 3 Centre for Neonatal Research and Education, Faculty Health and Medical Science, University of Western Australia, Perth, Western Australia, Australia; 4 School of Medicine, Division of Paediatrics, Faculty Health and Medical Science, University of Western Australia, Perth, Western Australia, Australia; 5 Perth Children’s Hospital, Perth, Western Australia, Australia; 6 School of Medicine, Otolaryngology Unit, Faculty Health and Medical Science, University of Western Australia, Perth, Western Australia, Australia; 7 Medical, Molecular & Forensic Sciences, Murdoch University, Perth, Western Australia, Australia; 8 Centre for Child Health Research, University of Western Australia, Perth, Western Australia, Australia; University of Pittsburgh, UNITED STATES

## Abstract

Recurrent and chronic otitis media (OM) are often refractory to antibiotics due to bacterial persistence in biofilm within the middle ear. *In vitro* and *in vivo* studies have demonstrated that antimicrobial proteins and peptides (AMPs) are bactericidal against otopathogens, indicating potential therapeutic value for recalcitrant OM. We measured concentrations of 6 AMPs and 14 cytokines in middle ear effusion (MEE) from 67 children undergoing ventilation tube insertion for recurrent acute OM. Sixty one percent of children had bacterial otopathogens detected in their MEE, 39% by PCR and 22% by PCR and culture. Groups were defined as: PCR-negative/culture-negative (absence of bacterial otopathogen), n = 26; PCR-positive/culture-negative (presence of nonculturable bacterial otopathogen), n = 26; PCR-positive/culture-positive (presence of culturable bacterial otopathogen), n = 15. Age, antibiotic usage, day-care attendance, presence of respiratory viruses in MEE and number of AOM episodes were similar between groups. AMP and cytokine concentrations were higher in children with bacterial otopathogens in their MEE compared to those with no bacterial otopathogens. Median concentrations of AMPs (except HBD2) were 3 to 56-fold higher in MEE from children with bacterial otopathogens detected in their MEE (*P* ≤ 0.01). Similarly, median cytokine concentrations (except TGFβ) were >16-fold higher in MEE with bacterial otopathogens detected (*P* ≤ 0.001). This is the first study to measure AMPs in MEE and together with the cytokine data, results suggest that elevated AMPs and cytokines in MEE are a marker of inflammation and bacterial persistence. AMPs may play an important role in OM pathogenesis.

## 1 Introduction

Otitis media (OM) is a common paediatric disease, and in high income countries OM is the primary reason that young children visit their General Practitioner, are prescribed antibiotics and undergo surgery [1]. Complications from recurrent and/or chronic OM include hearing loss and speech delay [2]. These sequelae contribute to a significant social and economic burden of OM on families and healthcare systems [3, 4]. Both bacteria and viruses are implicated in OM aetiology [5], with bacteria more often associated with chronic and recurrent OM [6]. The major bacterial species that are associated with OM are nontypeable *Haemophilus influenzae* (NTHi), *Streptococcus pneumoniae* and *Moraxella catarrhalis*, with NTHi being the most frequently detected species from the middle ear of children with recurrent and chronic OM [6]. Bacterial otopathogens can resist host immune responses and antibiotics through persistence mechanisms such as the ability to form and maintain biofilm [7, 8]. To reduce the global burden of OM, especially recurrent and chronic OM, new strategies that target bacterial otopathogens are urgently required.

Antimicrobial peptides and proteins (AMPs), otherwise known as host defence peptides, are rapid mediators of the innate immune system with broad-spectrum antimicrobial, antibiofilm and chemotactic activity that can influence the microbiota of mucosal surfaces [9–11]. There is compelling evidence from microbiological assays and animal models demonstrating direct and indirect roles for AMPs in OM pathogenesis [12–17]. For example, modulating the availability of AMPs has been shown to influence otopathogen clearance in the chinchilla model of OM. Reducing the availability of chinchilla beta-defensin 1 (equivalent to human beta-defensin 3) through RSV co-infection or with a blocking antibody resulted in increased NTHi loads from nasopharyngeal lavage fluids while increasing the availability of chinchilla beta-defensin 1, via delivery of a recombinant form, was associated with reduced NTHi loads [14]. Furthermore, in the *junbo* mouse model of OM, deletion of the gene encoding an AMP from the bactericidal permeability increasing (BPI) family (BPIFA1) resulted in exacerbation of OM severity due to increased epithelial remodelling [15]. Based on this, Mulay *et al* suggest that AMPs play a homeostatic role in maintaining healthy middle ear epithelium. Formulations of AMPs that could be applied intranasally or through the tympanic membrane (either via ventilation tubes, perforations or even through transmembrane adsorption) offer a potential antimicrobial therapy for children with recurrent and chronic OM, which may also reduce antibiotic use and curb the global rise in antibiotic resistance [10, 18, 19]. However, the direct measurement of AMPs in the middle ear of children with recurrent and chronic OM has not been reported before. This is critical to understand the relationship between AMPs and OM pathogenesis and to assess the potential use of these small molecules as therapeutic agents.

This study measured the concentrations of six AMPs in middle ear effusion (MEE) samples from children with a history of recurrent acute OM (rAOM). We hypothesised that children with no bacterial otopathogens detected in their MEE would have higher AMP titres than children with bacterial otopathogens in the MEE, due to the demonstrated antimicrobial activity of AMPs. We also measured a panel of fourteen cytokines in MEE from the same children to assess inflammatory responses and because there is important interplay between AMPs and pro-inflammatory cytokines particularly in epithelial cell and monocyte responses [17, 20, 21]. Cytokines, particularly those involved in the innate immune response, are integral for pathogen clearance and orchestrating down-stream adaptive responses. While there have been a number of earlier studies measuring middle ear cytokine responses in paediatric OM samples [22–25], studies were limited to measuring one or two cytokines in each cohort, most likely due to limited sample volume and use of ELISAs rather than multiplexed small volume bead-based immunoassays. Thus, we used multiplexed small volume bead-based immunoassays to measure a panel of 6 AMPs and 14 cytokines in MEE. We then assessed the concentrations of these important mediators in the context of otopathogen detection by comparing the following groups: 1) absence of bacterial otopathogen 2) presence of nonculturable but live bacterial otopathogen and 3) presence of culturable bacterial otopathogen in MEE, to understand the role of inflammation and innate immunity in the pathogenesis of recurrent OM.

## 2 Methods

### 2.1 Study cohort

This study used MEE samples collected from children enrolled in the GROMIT study [26]. All children had a history of rAOM and were undergoing ventilation tube insertion at the time of MEE collection. Age, gender, antibiotic usage currently and in the previous month before surgery, mean number of AOM episodes, day-care attendance (>4 hours per week), exposure to cigarette smokeand PCV vaccine status (received 3 doses of PCV7 or not) were recorded. Culture and PCR of MEE for the major bacterial otopathogens and respiratory viruses has been previously reported [26, 27]. Of the 186 children undergoing ventilation tube insertion in the original cohort, MEE was available for 67 children for this study due to sample volume remaining after primary outcomes were assessed. Of the 67 children in this study, questionnaire data was available for 59/67. This study was approved by the Ethics Committee of Princess Margaret Hospital for Children, Perth, Western Australia (EP1295) and by ethics committees and the institutional boards of the hospitals where recruitment took place. Written informed consent was obtained from parents of participating children before any study procedures were performed.

### 2.2 Collection, processing and storage of middle ear effusion

MEE samples were collected from anaesthetised children as previously described [26]. In brief, a sterile Argyle specimen trap (Covidien, Ireland) was connected to the surgical suction system to remove the MEE though the myringotomy incision. The tubing was rinsed with sterile saline to ensure that all MEE was collected. The MEE was placed on ice and transported to the laboratory within 4h where the sample was vortexed vigorously and 200μl transferred into media for bacteriological culture. The remaining MEE was transferred into 200μl aliquots and stored at -80°C for detection of respiratory viruses [27], and measurement of total protein [28], antibody [29], AMP and cytokine titres.

### 2.3 Quantification of AMP and cytokine concentrations in MEE

An aliquot of MEE was thawed and used to quantify the following AMPs: Human beta defensin 1 (HBD1), Human beta defensin 2 (HBD2), LL-37, lactoferrin, bactericidal/permeability-increasing protein (BPI) and secreted phospholipase A_2_ (sPLA_2_). HBD1, HBD2, LL-37 and lactoferrin levels were measured using in-house multiplex fluorescent bead-based assays on the Bio-Plex® 200 system (Bio-Rad Laboratories, California, USA) [30]. BPI and sPLA_2_ levels were measured using commercial ELISA kits (Hycult Biotech, Plymouth Meeting, PA, USA, and Cayman Chemical, Ann Arbor, MI, USA, respectively) and according to the manufacturers’ instructions.

Fourteen cytokines were quantified in another aliquot of thawed MEE: IL-1α, IL-1β, IL-5, IL-6, IL-8, IL-10, IL-12p70, IL-13, IL-17A, IFNγ, TNFα and TFGβ1, 2 and 3. IL-1β, IL-5, IL-6, IL-8, IL-10, IL-12p70, IL-13, IL-17A, IFNγ, and TNFα levels (pg/mL) were measured using an in-house multiplex cytokine bead assay on the Bio-Plex® 200 system (Bio-Rad Laboratories, California, USA) using previously published methods [31]. TFGβ1, 2 and 3 and IL-1α (pg/mL) levels were measured using commercial Luminex and ELISA kits respectively (R and D systems, Minneapolis, USA) according to the manufacturers’ instructions. Levels in pg/mL were generated from a seven-point, four and five-parameter logistic standard curves. AMP and cytokine levels that were below the limit of detection were assigned a value that was half of the lowest detectable amount of mediator specific for each assay.

Total protein was previously determined in each MEE sample using the Micro BCA Protein Assay Kit and according to the manufacturers’ instructions (Thermo Scientific, Rockford, IL, USA) [28]. Levels of AMPs and cytokines were normalised to total protein in MEE (pg of mediator/mg of protein). Samples that were below the limit of detection were reassigned a value that was the lowest concentration based on half the limit of detection normalised to total protein. Assigned limit of quantification were as follows: HBD1 = 2.84 pg/mg of protein; LL-37 = 507.8 pg/mg of protein; BPI = 12.45 pg/mg of protein; sPAL_2_ = 1.88 pg/mg of protein; IL-1β, IL-5, IL-12p70, IFNγ and TNFα = 0.09 pg/mg of protein; IL-6 and IL-10 = 0.57 pg/mg of protein; IL-8 = 3.17 pg/mg protein; IL-13 and IL-17A = 0.18 pg/mg of protein; TFGβ1 = 3.51pg/mg protein; TFGβ2 = 1.35pg/mg protein TFGβ3 = 5 pg/mg protein and IL-1a = 0.32 pg/mg protein.

### 2.4 Statistical analyses

Statistical analyses were performed using the IBM SPSS® software platform version 24. Population demographics were compared between the 3 groups of children: 1) PCR-ve/culture-ve, 2) PCR+ve/culture-ve and 3) PCR+ve/culture+ve, using Kruskal-Wallis tests for continuous variables (age, number of AOM episodes) and Pearson Chi-square analysis (*P*-value asymptotic significant 2-sided) for categorical variables (gender, day-care attendance, antibiotic usage, respiratory virus presence and bacterial otopathogen presence). Data were checked for normal distribution. AMP and cytokine levels were compared between PCR-ve/culture-ve; PCR+ve/culture-ve; PCR+ve/culture+ve children using Kruskal-Wallis tests with post-hoc pairwise comparisons for non-parametric data.

## 3 Results

### 3.1 Population demographics

The 67 children in this study were divided into three groups based on bacterial otopathogen detection of NTHi, *S*. *pneumoniae* and/or *M*. *catarrhalis* in MEE by PCR and culture as shown in Table 1. Groups included: PCR-negative/culture-negative (absence of bacterial otopathogen), n = 26; PCR-positive/culture-negative (presence of nonculturable bacterial otopathogen), n = 26; PCR-positive/culture-positive (presence of culturable bacterial otopathogen), n = 15. Median age, gender, recent and current antibiotic usage, mean number of AOM episodes and day-care attendance were similar across groups. The proportion of children on current antibiotics was not statistically different across groups. When current antibiotic usage was compared between children with PCR+ve/culture-ve MEE to those with PCR+ve/culture+ve MEE, there was a trend for fewer positive cultures from children who were on antibiotics at the time of ventilation tube insertion; *P* = 0.07). Ninety-seven percent of children had received all 3 doses of the 7-valent pneumococcal conjugate vaccine according to the Australian immunisation schedule. Nucleic acid from at least one of 11 common upper respiratory tract viruses was detected in 54 of the 67 children. The frequency of respiratory virus detection was similar between all 3 groups: PCR-ve/culture-ve = 73.1%, PCR+ve/culture-ve = 80.8% and PCR+ve/culture+ve 93.3% (Table 1). However, the proportion of children with respiratory virus in their MEE incrementally increased (not significantly) when bacterial otopathogen was detected by PCR and then by both PCR and culture. When bacterial otopathogen was detected by culture it was always detected by PCR. NTHi was the predominant otopathogen detected by PCR with 80.4% of children harbouring this pathogen. *M*. *catarrhalis* and *S*. *pneumoniae* were detected in 21.6% and 19.1% of children respectively (Table 1).

**Table 1:** Population demographics.

	PCR–ve/ culture-ve n = 26	PCR +ve/ culture-ve n = 26	PCR+ve/ culture+ve n = 15	p value
**Median age in months**	20.9	20.6	17.3	0.746
**Male gender, n (%)**	16 (61.5)	20 (76.9)	13 (86.7)	0.186
**Antibiotics used in the last month, n (%)^#^**	19 (79.2)	19 (82.6)	9 (75)	0.866
**Antibiotics currently, n (%)^#^**	9 (37.5)	11 (47.8)	2 (16.7)	0.194
Mean number of AOM episodes (total)*	7.9	6.9	6.6	0.930
**Day-care (4+hrs per week), n (%)^$^**	16 (69.6)	15 (68.2)	7 (58.3)	0.785
**Exposure to cigarette smoke, n (%)^#^**	7 (28)	4 (17.4)	1 (0.08)	0.310
**PCV7 vaccination (3 doses), n (%)**	25 (96.2)	26 (100)	14 (93.3)	0.456
**Presence of any respiratory virus, n (%)**	19 (73.1)	21 (80.8)	14 (93.3)	0.287
**Culture+ve for any otopathogen, n (%)**	0 (0)	0 (0)	15 (100)	>0.001
**Otopathogen detection in MEE by PCR:**				
**Any of the 3 otopathogens, n (%)**	0 (0)	26 (100)	15 (100)	>0.001
**NTHi, n (%)**	0 (0)	21 (80.8)	12 (80.0)	>0.001
***S*. *pneumoniae*, n (%)**	0 (0)	3 (11.5)	4 (26.6)	0.026
***M*. *catarrhalis*, n (%)**	0 (0)	6 (23.1)	3 (20.0)	0.036

^#^ n = 24/26 (PCR-ve/culture-ve); 23/26 (PCR+ve/culture-ve); 12/15 (PCR+ve/culture+ve)

* n = 24/26 (PCR-ve/culture-ve); 24/26 (PCR+ve/culture-ve); 12/15 (PCR+ve/culture+ve)

^$^ n = 23/26 (PCR-ve/culture-ve); 22/26 (PCR+ve/culture-ve); 12/15 (PCR+ve/culture+ve)

### 3.2 AMP levels in MEE from children with a history of rAOM were higher when bacterial otopathogen/s were also detected in their MEE

**Fig 1:**
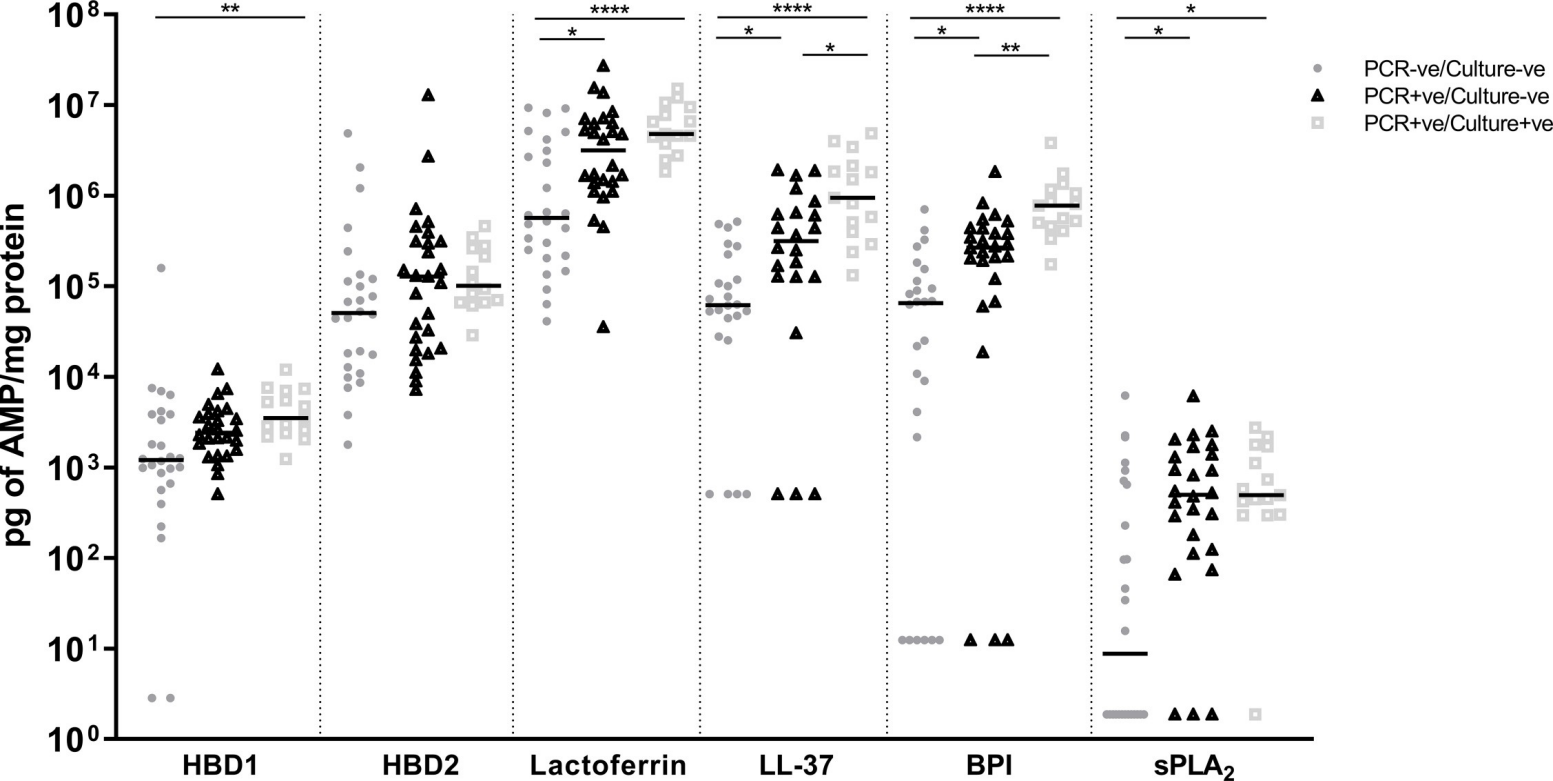
AMP levels in MEE from children with a history of rAOM were higher when bacterial otopathogen was also detected in MEE. Levels of HBD1, HBD2, lactoferrin, LL-37, BPI and sPLA_2_ in MEE normalised to total protein in children grouped according to bacterial otopathogen detection in MEE; PCR-ve/culture-ve (n = 26^#^); PCR+ve/culture-ve (n = 26^#^) and PCR+ve/culture+ve (n = 15). Line depicts median titres. ^#^LL-37; PCR-ve/culture-ve (n = 24/26); PCR+ve/culture-ve (n = 22/26). ****P ≤ 0.0001; ***P ≤ 0.001; **P ≤ 0.01; *P ≤ 0.05.

All of the 6 AMPs tested could be measured and most children had levels of each AMP above the limit of detection for each assay. Except for HBD2, median concentrations were significantly higher in MEE from children that had detectable bacterial otopathogen by PCR and were highest in children with otopathogen that could be detected in their MEE by both PCR and culture (*P* ≤ 0.01; Fig 1). Median concentrations of HBD1 were approximately 3-fold higher in PCR+ve/culture+ve MEE compared to PCR-ve/culture-ve MEE (*P* = 0.002). Median titres of lactoferrin were 9-fold higher in PCR+ve/culture+ve compared to PCR-ve/culture-ve MEE (*P* < 0.0001), and 6-fold higher in PCR+ve/culture-ve compared to PCR-ve/culture-ve MEE (*P* = 0.022). Median concentrations of LL-37, BPI and sPLA_2_ were 15-, 12- and 56-fold higher respectively in PCR+ve/culture+ve MEE compared to PCR-ve/culture-ve MEE (*P* < 0.0001; *P* < 0.0001; *P* = 0.012). LL-37 and sPLA_2_ levels were 5- and 56-fold higher (respectively) in PCR+ve/culture-ve MEE compared to MEE from children with no bacterial otopathogen detected (PCR-ve/culture-ve MEE), *P* = 0.018; *P* = 0.015. Median BPI levels were 4-fold higher in PCR+ve/culture-ve MEE compared to PCR-ve/culture-ve MEE (*P* = 0.015) and 3-fold higher in PCR+ve/culture+ve MEE compared to PCR+ve/culture-ve MEE (*P* = 0.006). Median concentrations of HBD2 were approximately 2-fold higher in PCR+ve/culture+ve MEE compared to PCR-ve/culture-ve MEE but this was not significant.

### 3.3 Pro-inflammatory cytokine levels in MEE from children with a history of rAOM were higher when bacterial otopathogen was also detected in their MEE

**Fig 2:**
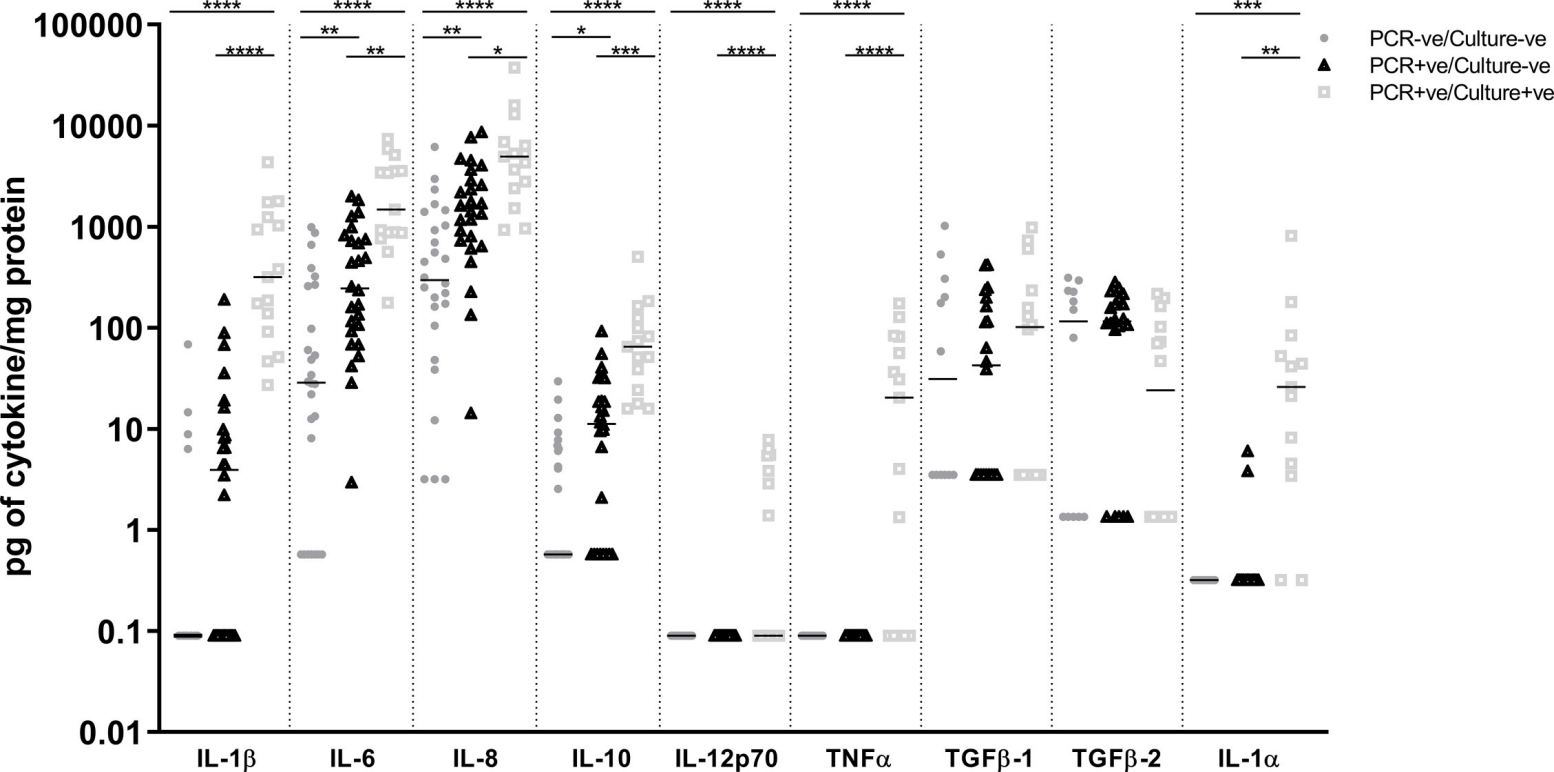
Cytokine levels in MEE from children with a history of rAOM were higher when bacterial otopathogen was also detected in MEE. Levels of IL-1β, IL-6, IL-8, IL-10, IL-12p70, TNFα, TGFβ-1, TGFβ-2, TGFβ-3 and IL-1α, in MEE normalised to total protein in children grouped according to bacterial otopathogen detection in MEE; PCR-ve/culture-ve (n = 26^#^), PCR+ve/culture-ve (n = 26^#^) and PCR+ve/culture+ve children (n = 15^#^). Line depicts median titres. ^#^only a subset from each group had enough MEE left to measure TGFβs: PCR-ve/culture-ve (n = 12/26); PCR+ve/culture-ve (n = 20/26) and PCR+ve/culture+ve (n = 14/15) and IL-1α: PCR-ve/culture-ve (n = 8/26); PCR+ve/culture-ve (n = 14/26); PCR+ve/culture+ve (n = 13/15). ****P ≤ 0.0001; ***P ≤ 0.001; **P ≤ 0.01; *P ≤ 0.05.

IL-5, IL-13, and IFNγ were not detected in MEE and IL-17A was only detected in 1 child (7.1 pg/mg of protein). Concentrations of IL-1α, IL-1β, IL-6, IL-8, IL-10, IL-12p70 and TNFα were always significantly higher in MEE from children when bacterial otopathogen was detected by culture and PCR compared to when bacterial otopathogen was not detected (*P* < 0.001; Fig 2). Concentrations of IL-1α, IL-1β, IL-6, IL-8 and IL-10 were also at least 3-fold higher in MEE from children when bacterial otopathogen was detected by both PCR and culture (PCR+ve/culture+ve) compared to PCR only (PCR+ve/culture-ve; *P* ≤ 0.031). IL-1β, IL-6, IL-8 and IL-10 levels were at least 5-fold higher in PCR+ve/culture-ve MEE compared to PCR-ve/culture-ve MEE (*P* ≤ 0.044). IL-12p70 and TNFα were only measurable in 7/67 and 10/67 children respectively but these children all had detectable bacterial otopathogen in their MEE by both PCR and culture. Similarly, IL-1α was only measureable in 13/35 children but these children all had bacterial otopathogen detected by both PCR and culture or PCR alone. TGFβ3 was not detected in MEE while concentrations of TGFβ1 and TGFβ2 were similar across groups (Fig 2).

## 4 Discussion

In this study, we measured for the first time, a suite of 6 AMPs and 14 cytokines in MEE from children undergoing ventilation tube insertion for rAOM. AMP and cytokine concentrations were compared between children that had bacterial otopathogens present in their MEE compared to those who did not. While children with a history of rAOM do not undergo surgery during an acute episode of OM, and have often been on antibiotics within a month prior to or during surgery, a high proportion of children still have live (sometimes culturable) bacterial otopathogens in their MEE. Contrary to our hypothesis, levels of AMPs and cytokines were lowest in MEE from children with no bacterial otopathogen detected; while higher AMP and cytokine titres were observed in MEE from children with bacterial otopathogen detected by PCR, and the highest titres were found in MEE from children with bacterial otopathogen detected by both PCR and culture. This indicates endogenous AMP levels are not adequate to clear bacterial otopathogen or that bacterial persistence mechanisms are at play (e.g biofilms). This has implications for helping to understand rAOM pathogenesis and suggests that elevated concentrations of AMPs and pro-inflammatory cytokines in MEE may be a marker of inflammation associated with bacterial persistence and/or presence of neutrophil extracellular traps (NETs), which are decorated in AMPs [32].

OM pathogenesis is complex and multifactorial. Nasopharyngeal and middle ear biofilms are increasingly recognised as playing a central role in OM pathogenesis by providing a bacterial reservoir, enabling bacterial persistence and recurring or chronic infection [7, 33]. PCR is widely accepted as superior for detecting bacterial otopathogens in the middle ear as traditional culture techniques often return false-negative results due to bacteria surviving in non-culturable but viable states [34]. It should be noted that while PCR will also detect DNA from dead bacteria, it has been shown with live/dead staining that otopathogens are usually viable in culture-negative MEE [8]. Furthermore, it has been shown in animal models that DNA from dead bacteria is rapidly cleared from MEE [35]. Our data certainly reflect this with only 15 out of 41 children with PCR positive MEE having bacterial otopathogens cultured from their MEE. We speculate that children with culture-positive MEE likely had planktonic bacteria, released from biofilm, and this represented a state of active infection [36]. This may explain why these children also had the highest concentrations of AMPs and pro-inflammatory cytokines in their MEE.

Our finding, that a greater inflammatory cytokine response was associated with bacterial otopathogen detection, particularly NTHi, is supported by several studies. Firstly, in adults with chronic obstructive pulmonary disease (COPD), higher levels of LL-37, IL-8, TNFα and greater inflammatory infiltrate were found in sputum with culturable NTHi compared to culture-negative sputum [37]. Secondly, LL-37 and lactoferrin levels in serum and sputum from adults with bronchiectasis were higher when *Pseudomonas aeruginosa* and NTHi were also present in sputum [38]. Thirdly, *in vitro* assays using chinchilla middle ear epithelial cells have revealed that NTHi challenge results in upregulation of mRNA for the chinchilla equivalent of LL-37, cCRAMP, which has bactericidal activity against the three major otopathogens [13]. Finally, in children followed over an AOM episode, higher levels of IL-8 were associated with otopathogen culture-positive MEE and these levels dropped after bacterial eradication with antibiotic therapy [25]. Together these data demonstrate the important and ongoing contribution of innate immune responses to invading pathogens, specifically NTHi, in the middle ear of children with a history of rAOM. In order to understand the relationship between AMP and cytokine concentrations and otopathogen clearance from the middle ear, longitudinal studies using tympanocentesis during active and convalescent stages of OM and complementary *in vitro* and *in vivo* models are required [14, 39, 40].

Previous studies have implicated the TGF-β pathway and IL-1α actions in OM pathogenesis. In rat models of OM, microarray experiments have revealed that bacterial OM rather than Eustachian tube obstruction drives TGF-β signalling. Furthermore, NTHi OM rather than *S*. *pneumoniae* OM resulted in more potent middle ear granulation tissue formation [41]. In a large Western Australian cohort study of non-Aboriginal children, single nucleotide polymorphisms within FBX011, a gene that regulates TGF-β signalling, have been associated with severe OM [42]. *In vitro* studies have demonstrated that NTHi induces the secretion of IL-1α by human middle ear epithelial cells and that this acts synergistically with NTHi to upregulate HBD2 mRNA transcription [17]. Surprisingly, in our analysis, levels of TGF-β1, 2 and HBD2 were similar between all children and there was no association with bacterial otopathogen detection. However, HBD2 levels in MEE tended to be higher in children when otopathogen was also detected and this corresponded with significantly higher levels of IL-1α. We acknowledge that these unexpected results could be due to the fact that TGF-β1, 2, and 3 were measured in MEE from a small number of children in this study and thus not powered enough to see small differences. However, it is also possible that the TGF-β pathway is not a key player in children with a history of rAOM with potent inflammatory responses and perhaps, as Mulay *et al*., suggest TGF-β is more important in epithelial remodelling after resolution of inflammation [15].

The higher concentrations of AMPs and inflammatory cytokines associated with bacterial otopathogen detection in our study may be indicative of more severe or persistent disease and the presence of non-resolving NETs [8]. Although it should be noted that children with otopathogen negative MEE had some degree of clinical inflammation otherwise it would not have been possible to obtain MEE from these children. This could be due to other species of bacteria that were not measured or from viral pathogen exposure, as nucleic acid from common upper respiratory tract viruses were frequently detected in MEE from children without bacterial otopathogen. It is also well known that respiratory viruses can cause AOM in the absence of bacterial co-infection [5]. Even in the few studies where cytokines have been measured in MEE from children with OM in which bacterial versus viral aetiology was not determined, high levels of TNFα and IL-1β were correlated to more chronic OM [23, 24]. Furthermore`, higher levels of TNFα were found in MEE from children who had undergone repeat ventilation tube insertion [23], suggesting these children had more severe and recurrent disease. Similarly, in adults with COPD, higher levels of sputum LL-37, IL-8, TNFα, and a greater proportion of neutrophils were predictive of episodes of acute exacerbated COPD, the more severe form of COPD [37]. Most notably, resolution of inflammation following episodes of acute COPD exacerbation were associated with reduction or eradication of bacteria [43]. This may also hold true for children with recurrent and chronic OM where elimination of pathogenic bacteria from the middle ear will likely resolve inflammation, reduce disease chronicity and potentially even impact the high need for repeat ventilation tube insertion [44]. Thus, eliminating bacterial reservoirs from the middle ear of children with recurrent and chronic OM should be a major and continuing treatment focus.

Whether elimination of bacterial otopathogensfrom the middle ear of children could be achieved through application of AMP doses that exceed physiological levels through ventilation tubes as an adjunct to traditional antibiotic treatment remains elusive. Preclinical models have provided critical proof-of-concept data for the therapeutic application of AMPs. Synergy between synthetic AMPs and antibiotics have been more effective than antibiotics alone for reducing the severity of cutaneous abscesses in a murine infectious abscess model [45]. In chinchillas with experimental OM, NTHi density in the nasal passages, which confers OM development, could be reduced via intranasal application of a recombinant chinchilla beta-defensin 1 (cBD1) as well as the human ortholog HBD3, demonstrating the capacity of these innate molecules to clear bacterial otopathogen [14]. However, there are several caveats that need to be considered before AMPs are used as an adjunct therapy for children with recurrent and chronic OM. Firstly, the levels of AMPs that would be required to clear bacterial otopathogens in children are not known and concentrations of synthetic AMPs needed for anti-biofilm/anti-microbial activity possibly exceed endogenous levels [46]. Secondly, potential off-target or ototoxic effects of AMPs could have deleterious consequences [47]. Thirdly, the presence of middle ear bacterial biofilms and particularly NTHi biofilms could render AMPs inactive. This could be through binding of AMPs to extracellular DNA [48, 49] in NETs, which are a key component of middle ear biofilm [8]. Alternatively, AMPs could be rendered inactive through upregulation of bacterial virulence genes to prevent killing from AMPs and to restore potassium homeostasis allowing otopathogens to survive in the middle ear [50–52]. These bacterial persistence mechanisms may be overcome by using AMPs in combination with anti-biofilm agents such as DNAses [8] or with NTHi vaccines that target biofilm antigens to release the bacteria from biofilm and increase their susceptibility to antimicrobial agents [53]. These potential strategies warrant further investigation.

In conclusion, we have provided the first details on the presence and biological range of AMPs that are present in MEE from children with a history of rAOM. We have also shown that AMPs, pro-inflammatory cytokines and the regulatory cytokine IL-10 are associated with presence of bacterial otopathogens in the middle ear. To reduce the global burden of recurrent and chronic OM, new therapies that focus on eradicating bacterial otopathogens from the middle ear to reduce inflammation are urgently needed. Whether this can be achieved through the therapeutic use of AMPs requires further research, but our data provide important insight into the physiological concentrations of AMPs which may need to be exceeded in a therapeutic setting.

## 5 Limitations

We acknowledge that our study has some minor limitations predominantly due to sample size and sample volume constraints. Due to the rarity of this sample type and volume needed for immune and microbiological assays, there were small numbers in this study. This may have been a contributing factor to the similar levels of TGFβ observed between groups when numbers were further restricted. Additionally, due to volume limitations, we did not quantify immune cells, specifically neutrophils, in MEE from children in this study. However, we have previously identified neutrophil infiltrate and NETs in MEE from other children in this cohort [8], thus, higher levels of AMPs and cytokines in otopathogen-positive MEE may also reflect greater inflammation due to recruitment of immune cells into the middle ear of children with persisting bacterial otopathogen.
